# Positive COVID19-PCR patients as negative controls for COVID19 antibody tests

**DOI:** 10.1016/j.amsu.2021.102320

**Published:** 2021-04-19

**Authors:** Nasr Alrabadi, Razan Haddad, Ibrahim Al-Faouri, Nail Obeidat, Mohammad Al-Ghazo, Khalid El-Salem, Ziad Elnasser, Nizar Mhaidat

**Affiliations:** a Department of Pharmacology, Faculty of Medicine, Jordan University of Science and Technology, Irbid, 22110, Jordan; b Department of Pharmaceutical Technology, Faculty of Pharmacy, Jordan University of Science and Technology, Irbid, 22110, Jordan; c Department of Community and Mental Health Nursing, Faculty of Nursing, Jordan University of Science and Technology, Irbid, 22110, Jordan; d Department of Obstetrics and Gynecology, Faculty of Medicine, Jordan University of Science and Technology, Irbid, 22110, Jordan; e Department of Surgery and Urology, Faculty of Medicine, Jordan University of Science and Technology, Irbid, 22110, Jordan; f Department of Neuroscience, Faculty of Medicine, Jordan University of Science and Technology, Irbid, 22110, Jordan; g Department of Pathology and Microbiology, Faculty of Medicine, Jordan University of Science and Technology, Irbid, 22110, Jordan; h Department of Clinical Pharmacy, Faculty of Pharmacy, Jordan University of Science and Technology, Irbid, 22110, Jordan

**Keywords:** COVID-19, Immunity, Negative control, Positive control, Antibody, Seroconversion

## Abstract

COVID-19 serological antibody tests are recently needed for a relatively quick, affordable, and valuable assessment of the immunity toward COVID-19 infection. Furthermore, they can help with evaluating the sufficiency of the vaccination process and its longevity. There are limitations in the current approach of choosing the positive and negative control samples for the validation of those tests. Herein, we are proposing the use of blood samples from positive COVID-19 patients, at the beginning of the disease course, as negative control blood samples for the antibody tests. For more precision, both the negative and the positive control samples can be obtained from the same patients where the accuracy of the test will depend on its ability to detect the seroconversion, from negative to positive antibodies detection, within the same patient. Furthermore, when the validation of the test is accompanied by detecting/sequencing the viral genome in those COVID-19 patients, this can also aid in determining the accuracy of the test in detecting the immune response to specific viral variants. The latter notion is needed for the proper management of the COVID-19 crisis, new vaccines' manufacturing, and evaluating the vaccines' efficiencies. Finally, this approach could be requested/formulated by the regulatory agencies as part of the tests’ validation and can be “in-house” obtained by health facilities before its clinical use.

COVID-19 serological antibody tests are recently needed for a relatively quick, affordable, and valuable assessment of the immunity toward COVID-19 infection. Furthermore, they can help with evaluating the sufficiency of the vaccination process and its longevity [1]. The importance of such assessments needs valid and precise serological tests with proper positive and negative controls. To our knowledge, the manufacturing companies collect blood samples from previously infected patients to pool their positive control groups. Those positive control patients should have a positive COVID19-PCR test (or NAAT) and the blood samples are drawn after a sufficient post-infection period to give the human body enough time to produce COVID-19 specific antibodies. On the other hand, the negative control groups represent serological samples either collected and stored before the COVID-19 pandemic or taken from those who tested negative for COVID-19 using the PCR/NAAT techniques. More details about this process can be found on the FDA website [2].

There are limitations of having accurate positive control samples giving the hardship of determining an accurate definition for the confirmed COVID-19 cases among different countries and agencies, including the CDC and the WHO. Some with defining those cases as positive COVID19-PCR cases and others by accompanying the diagnostic tests with specific signs and symptoms. The positive COVID-19 cases, especially for the serological tests, could be determined more accurately through proper assessments of multifactorial immunological biomarkers including the activation of specific immune cells and specific immunological responses to COVID-19. Those assessments can be better conducted by manufacturing companies before releasing any new serological antibody kits. As such, those studies better be formulated and encouraged/requested by the regulatory agencies that approve the clinical use of those kits such as the FDA and EMA. This is necessary as it seems difficult and not practical to consider this approach by investigators while optimizing the use of those kits before conducting their studies or before the clinical use of the kits in health facilities.

Regarding the negative control groups and away from those collected before the COVID-19 pandemic (will not be discussed in this report), the approach which depends on considering the patients with negative COVID19-PCR test (and with negative history for COVID-19 infection) as the negative control group may be inaccurate. Unfortunately, most of the COVID-19 patients will never show signs or symptoms of the infection and their COVID19-PCR tests will become negative in less than a month. Such a scenario increased the risk of considering some of the patients valid for donating negative control blood samples while, in fact, their samples may contain COVID-19 specific antibodies because of previous asymptomatic COVID-19 infection. Therefore, this approach may jeopardize the accuracy of those kits. Furthermore, the high rate of false-negative results of the PCR tests (may reach up to 50%) can reduce the accuracy of this test to determine the negative control group [3].

Herein, we propose to use the blood samples of the patients with positive COVID19-PCR test and who are obviously sick and presented with the disease-specific signs and symptoms as negative control samples for validating the COVID-19 antibody kits. Those negative control blood samples should be collected at the beginning of the disease course (before the beginning of showing the specific signs and symptoms for the disease) and before giving the human body the chance to produce the neutralizing antibodies (either IgG or IgM according to the test purpose) [4]. As such, those samples can also be retrospectively evaluated as valid samples. This approach is valid assuming that there is no possibility of COVID-19 re-infection. However, even if such a possibility does exist, the current scientific evidence suggests that it is very low if any (for the same viral variants), and it can be considered very rare in comparison to the possibility of having a negative COVID19-PCR test in patients who previously infected and immunized but were asymptomatic and never diagnosed. Moreover, even if the patient suffered from re-infection, this favorably means that the previous infection is not sufficient to generate an immune response and neutralizing antibodies to prevent the re-infection. Therefore, the blood samples from those patients may still be valid as negative controls especially when using the positive signs and symptoms to define the infected cases (assuming that those signs and symptoms may only happen if the immunity or the neutralizing antibodies are critically low or negative).

Luckily, such an approach can be conducted in the research labs and the clinical facilities. Therefore, investigators can test and validate the currently available antibody kits in the market before conducting a research study or before their clinical use on patients. This in-house approach is important giving the differences in the test performance among different countries or ethnicities [5]. However, the manufacturing companies should be encouraged to adopt such approaches. As well, it is better for the regulatory agencies, such as the FDA or EMA, to formulate those approaches in their approval criteria and guidelines. Furthermore, it is important to note that those negative control samples can be taken fresh (in real-time) from the donors and not depending on old frozen samples. As well, other biomarkers can be detected and tested for those patients at the same time to increase the accuracy and validity of the tests.

Moreover, it can be recommended to go further with increasing the accuracy and the precision of those kits by collecting the negative control and the positive control samples from the same patients. The negative control samples can be collected at the beginning of the disease (when the COVID19-PCR test is positive at the beginning and before the appearance of the disease-specific signs and symptoms) and the positive control samples can be collected from the same patient after a specific post-infection period. According to this approach, the test can be considered valid if started negative then turned positive (a seroconversion happened) in the same patient.

On the other hand, it may be necessary, especially recently with the possibility of re-infection by new viral variants, to determine the viral variants in those infected patients and to examine the accuracy of the tests for specific variants of the SARS-CoV-2 virus. Specific kits for specific COVID-19 viral variants may become necessary soon for the test to be valid in determining the immunity after the COVID-19 infection or after the vaccination process. As such, this can also aid in determining the accuracy of the test in detecting if cross-immunity exists and therefore to design and evaluate the efficiency of the vaccines more accurately.

Finally, this approach is generalizable to other infectious diseases including other viral infections. In this report, we focused on the COVID-19 because of the seriousness of the current pandemic.

## Conclusions and remarks

This report highlights the importance of proper validation of the tests that are used for detecting the SARS-CoV-2 specific antibodies in the blood of the COVID-19 patients. In this regard, we proposed the use of blood samples from positive COVID-19 patients, at the beginning of the disease course, as negative control blood samples for the antibody tests. The ability of the test/kit to detect the antibodies seroconversion in the blood samples of COVID-19 patients is critical in validating their use. At this stage of having different variants of the SARS-CoV-2 virus, detecting/sequencing the viral genome in the same COVID-19 patients can also aid in determining the accuracy of the test in detecting the immune response to specific viral variants and to improve the vaccines manufacturing. Finally, we recommend formulating and requesting such approaches by the regulatory agencies as part of the tests’ validation by the manufacturing companies.

## Provenance and peer review

Not commissioned, externally peer reviewed.

## Ethical approval

As a short communication representing the authors' opinion about public issues, there was no need for ethical approvals.

## Sources of funding

This work is part of the COVID-19 project supported by the Deanship of Research at 10.13039/501100004035Jordan University of Science and Technology (Grant No. 20210019).

## Author contribution

All authors contributed significantly to preparing the manuscript. All authors presented substantial contributions and participated in the idea generation, corrections, and the final approval of the version to be submitted.

## Conflict of interest

The authors declared no conflict of interest.

## Registration of research studies

Not applicable for this type of studies.

## Guarantor

Dr. Nasr Alrabadi Nnalrabadi@just.edu.jo.
